# Regulation of chloroplast primary metabolism

**DOI:** 10.1007/s11120-020-00765-4

**Published:** 2020-06-14

**Authors:** Ute Armbruster, Deserah D. Strand

**Affiliations:** 1 Group “Regulation of Photosynthesis”, Max Planck Institute of Molecular Plant Physiology, Wissenschaftspark Golm, Am Mühlenberg 1, 14476 Potsdam, Germany; 2 Group “Organelle Biology and Biotechnology”, Max Planck Institute of Molecular Plant Physiology, Wissenschaftspark Golm, Am Mühlenberg 1, 14476 Potsdam, Germany

Land plants are sessile, and therefore cannot escape stressful conditions. They have, however, evolved a tremendous capacity to remodel their metabolism in order to cope with short and long-term environmental perturbations. Plants use light as the energy source to drive their metabolism. These photosynthetic reactions take place in the chloroplast, an organelle of prokaryotic origin. Chloroplast reactions include the thylakoid localized light reactions of photosynthesis, which produce oxygen and the energy equivalents ATP and NADPH via the concerted action of the two photosystems, photosystem I (PSI) and photosystem II (PSII), the cytochrome *bf* complex, and the ATP synthase; the carbon fixation reactions of the Calvin Benson Bassham (CBB) cycle, which use these energy equivalents to fix CO_2_; and the reactions that convert the output of the CBB cycle into starch for storage. These reactions occur in a quasi-linear manner from light energy transduction into metabolic energy, then into high-energy carbon bonds and finally their storage as high molecular “energy-rich” starch molecules. This chain of molecular events is interconnected by regulatory mechanisms, which are switched on upon perturbation to avoid the production of harmful side products such as reactive oxygen species. By increasing our understanding of the regulation of chloroplast primary metabolism in response to environmental stresses, we hope to contribute to new strategies for enhancing photosynthesis under adverse environmental conditions.

This Special Issue focuses on the regulation of the chloroplast energy conversion and storage pathways. Environmental stresses affect chloroplast energy transduction in a variety of ways. This Special Issue features publications that address three of the most frequent abiotic stresses that plants encounter in nature: temperature, light, and osmotic stress.

Temperature is a major determinant for the rate of enzymatic reactions and non-enzymatic side reactions. Changes in growth temperature trigger acclimatory long-term responses, which re-balance reactions to favor efficient metabolism. However, the mechanisms by which temperature acclimation achieves balanced primary metabolism in the chloroplast remain largely unclear. To address this gap in knowledge, Herrman et al. (2019) generated a computational model, which predicts metabolic changes in response to temperature acclimation. They then tested this model against light response curves generated from plants acclimated to different growth temperatures. Their model suggests that long-term acclimation to elevated temperatures, and interestingly, long-term cold stress, both lead to an upregulation of NADPH utilization. However, in cold stressed plants, this is accompanied by an increased capacity to fix carbon, while this capacity significantly decreased in plants acclimated to elevated temperature.

Light quantity and quality directly interact with the light reactions of photosynthesis. One regulatory mechanism to counteract light stress is the redistribution of light energy between the two photosystems via the lateral migration of PSII light harvesting complexes (LHCII). Both phosphorylation and acetylation have been shown as posttranslational modifications involved in the lateral re-distribution of LHCII complexes, a process referred to as state transitions (Bellafiore et al. 2005; Koskela et al. 2018). The responsible enzymes for the posttranslational modifications that trigger movement of LHCII from PSII to PSI are the thylakoid kinase STN7 (Bellafiore et al. 2005) and the chloroplast NSI acetylase (Koskela et al. 2018). In this issue, Koskela et al. show that mutants devoid of STN7 and NSI show very similar growth and photosynthetic defects under fluctuating light conditions. This finding highlights that LHCII phosphorylation, thylakoid protein acetylation by NSI and thus state transitions are important for plant photosynthesis under dynamic light environments. Additionally, Koskela et al., found an increased abundance of the one helix protein 1 (OHP1) in PSII dimers of the *nsi* mutants as compared to wild-type and *stn7*. OHP1 has a described function as an auxiliary factor of an early stage of PSII assembly (Li et al. 2019; Myouga et al. 2018). The function of OHP1 in PSII dimers of *nsi* mutants remains to be determined.

A posttranslational modification that responds directly to the redox state of the chloroplast and is strongly linked to light intensity is the formation of disulfide bridges. Intra-molecular disulfide bridge formation causes the deactivation of many chloroplast enzymes including those of the CBB cycle and the electron transport chain (reviewed in Kaiser et al. 2019). 2-Cys peroxiredoxin (2-CysPRX) acts as a thioredoxin oxidase and thereby accelerates the oxidative deactivation of chloroplast thioredoxin targets in response to shading (Vaseghi et al. 2018; Ojeda et al. 2018). In this Special issue, Telman et al. evaluate the role of two additional peroxiredoxins, PRXQ and PRXIIE as potential thioredoxin oxidases. They convincingly demonstrate that the thylakoid localized PRXQ also functions in this manner, albeit to a lesser extent than 2-CysPRX. PRXIIE instead does not act as a thioredoxin oxidase and its function in chloroplast redox homeostasis remains enigmatic.

Osmotic stress in plants can occur from soil salinity or leaching. In the chloroplast, envelope K^+^ exchange antiporters (KEA1 and KEA2) have been associated with osmotic regulation, as they have been shown to be crucial for regulating the ion homeostasis of the plant and thus chloroplast development and function (Aranda-Sicilia et al. 2016; Kunz et al. 2014). Interestingly, these proteins are post-translationally modified by acetylation and lack this modification in the absence of the NSI enzyme that is also responsible for state transitions (Koskela et al. 2018). The NSI acetylation sites of KEA1 and KEA2 are located within the N-terminus of their mature protein sequences. Another potential regulatory feature of KEA1 and KEA2 is located in their C-termini, which harbor a K^+^ transport nucleotide binding (KTN) domain. The KTN domain has been shown to regulate the activity of K^+^ transporters and channels in response to nucleotide levels (Cao et al. 2013; Kröning et al. 2007; Roosild et al. 2002). Bölter et al. in this issue, show that both regulatory features of KEA1/2, acetylation sites in the N-terminus and the KTN domain in the C terminus are exposed to the stroma. This finding supports regulation of KEA1/2 activity by acetylation via the stroma localized NSI enzyme and stromal nucleotide concentrations.

Starch allows the storage of energy derived from photosynthesis in an osmotically inert form. It is conceivable that the regulation of starch synthesis and degradation rates buffers the effects that environmental perturbations and resulting changes in energy conversion efficiencies would otherwise have on chloroplast osmotic homeostasis. Currently, we are still lacking a clear understanding of how starch granules derive their complex and osmotically inert structure. Pfister et al. summarize the experimental evidence for starch granule formation and argue, based on their own computational approach, for an increased synergy between experimentation and simulation to achieve a deep understanding of the molecular processes that underlie starch formation.

This Special Issue addresses chloroplast primary metabolism and several of the regulatory processes that underlie its response to environmental perturbation and bridges photosynthetic energy conversion with subsequent storage in the chloroplast. The presented publications (i) uncover novel regulatory players on protein (PRXQ, (Telman et al. 2019) and on metabolic level [NADPH and CO_2_ utilization, (Herrmann et al. 2019)], (ii) reveal potential regulation strategies by stromal factors (Bölter et al. 2019), (iii) support the important role of posttranslational modifications for dynamic photosynthesis (Koskela et al. 2020) and (iv) underline the function of biosynthetic enzymes and glucan secondary structures in starch architecture (Pfister et al. 2020).
